# Obesity in Postmenopausal Breast Cancer Patients: It Is Time to Improve Actions for a Healthier Lifestyle. The Results of a Comparison Between Two Italian Regions With Different “Presumed” Lifestyles

**DOI:** 10.3389/fonc.2021.769683

**Published:** 2021-11-18

**Authors:** Laura Cortesi, Giulia Raffaella Galli, Federica Domati, Luana Conte, Luigi Manca, Maria Antonietta Berio, Angela Toss, Anna Iannone, Massimo Federico

**Affiliations:** ^1^Department of Oncology and Hematology, University Hospital of Modena, Modena, Italy; ^2^Breast Unit City of Lecce Hospital, Gruppo Villa Maria (GVM) Care & Research, Lecce, Italy; ^3^Department of Medical and Surgical Sciences, University of Modena and Reggio Emilia, Modena, Italy; ^4^Laboratory of Biomedical Physics and Environment, Department of Mathematics and Physics, University of Salento, Lecce, Italy; ^5^Laboratory of Interdisciplinary Research Applied to Medicine, University of Salento and Azienda Sanitaria Locale (ASL), Lecce, Italy; ^6^Department of Surgery, Medicine, Dentistry and Morphological Sciences with Transplant Surgery, Oncology and Regenerative Medicine Relevance, University of Modena and Reggio Emilia, Modena, Italy

**Keywords:** body mass index, breast cancer, Mediterranean diet, overweight, obesity

## Abstract

**Background:**

Adult body fatness is a convincing risk factor for postmenopausal breast cancer. With the aim to compare the different breast cancer (BC) features in Northern and Southern Italy, we investigated the relationship between BMI and BC characteristic in two groups of patients referred in the Modena and Lecce breast units.

**Materials and Methods:**

A retrospective analysis of a continuous series of BC patients referred to the Città di Lecce Hospital and the Modena Cancer Center, from January 2019 to December 2020 was performed. We identified four groups of BMI at BC diagnosis: underweight, BMI <18.5 kg/m^2^; normal weight, BMI ≥ 18.5–24.9 kg/m^2^; overweight, BMI ≥ 25.0–29.9 kg/m^2^; obese, BMI ≥30.0 kg/m^2^. BC characteristics and clinical outcomes were analyzed by the Kolmogorov-Smirnov test and Mann-Whitney *U* test; categorical data were compared using Pearson’s chi-square test, and dicotomic data were compared by odds ratio.

**Results:**

Nine hundred seventy-seven BC patients were included in the analysis. Overall, 470 were from Modena and 507 from Lecce. No differences were observed in the mean age of BC patients of Modena (61,42) and Lecce (62,08). No statistical differences between the two populations were shown in terms of tumor characteristics and pathological stage. Conversely, a statistical difference of BMI between the BC patients coming from Modena and Lecce (25.87 and 27.81, respectively; *p* = 0.000001) was found. BC patients diagnosed in Lecce at age ≥70 years had higher median BMI compared with the ones from Modena (*p* = 0.000002). The increased BMI in this aged population was also associated to larger tumor size (*p* = 0.040).

**Conclusion:**

The rate of overweight and obesity was higher in BC women living in Southern Italy, despite the presumed nutrition according to the so-called Mediterranean type dietary pattern. Unexpectedly, an increased BMI rate and a relationship with larger tumor size were found in Southern BC patients aged ≥70 years. Our findings strongly support the need for promoting a healthier lifestyle model in Italy, with the aim of reducing the rate of the obesity and, consequently, the increased risk of BC.

## Introduction

Breast cancer (BC) is the most frequent malignancy in women worldwide. In the USA, it is estimated that there are currently 3.8 million breast cancer survivors (1), while in Italy, in 2020, breast cancer reached 13% of the whole population (2). In Italy, geographical comparisons were conducted based on AIRTUM 2008–2016 data, highlighting a North-South geographical gradient in breast cancer incidence affecting the female population. In this contest, BC level decrease from the Northern (162.6/cases/year/100,000) to the Southern and islands (123.6/cases/year/100,000) (3). There are numerous risk factors such as sex, aging, hormonal factors, family history, genetic predisposition, and unhealthy lifestyle, increasing the possibility of developing BC (4). In particular, the excessive body weight is one of the most important contributors to mortality worldwide. Indeed, overweight has been associated with elevated risks of many chronic diseases (5), and it is considered an established risk factor for several cancer types (6, 7), including BC. The evidence shows that high body mass index (BMI), which is usually used to define overweight or obese people, has significant positive associations with breast cancer risk among postmenopausal women (7). As adipose tissue is a source of estrogens, one plausible mechanism by which postmenopausal obesity increases the risk for developing breast cancer is through higher levels of endogenous estrogen (8). In postmenopausal women, increased levels of estrone, estradiol, and free estradiol are related to BMI (9–16). This relationship may be modified by physical activity (PA) resulting in lower serum levels of estrogens (9). Consequently, a healthy lifestyle is related to a reduced risk of BC, especially in postmenopausal women. On the contrary, studies in premenopausal women are conflicting. Some studies have shown a decreased risk for premenopausal breast cancer, while others have suggested no association (17). The American Institute for Cancer Research (AICR) and World Cancer Research Fund (WCRF) continuous updated report on nutrition and cancer (18) currently defines “adult body fatness” as a convincing risk factor for postmenopausal breast cancer and as a probably decreasing breast cancer risk for premenopausal women.

Innumerable health benefits accompany favorable lifestyle modification, and such changes are at very low risk and unexpensive. Many guidelines and recommendations have been released (18), suggesting having healthy lifestyle and behavior, but they are not often followed, as demonstrated by the obesity epidemic of our country (2). A simple modality to maintain a healthy lifestyle is represented by the so-called Mediterranean-type dietary pattern. There are several variants of the Mediterranean diet, but some common components can be identified: high monounsaturated saturated fat ratio; ethanol consumption at moderate levels and mainly in the form of wine; high consummation of vegetables, fruits, legumes, and grains; moderate consumption of milk and dairy products; and low consumption of meat and meat products. The Mediterranean diet can be considered a lifestyle that include the importance of sleep, the effects of happiness and other psychological aspects on longevity, and the relationship between PA, exercise, and health (19). A protective role of the Mediterranean diet on BC is biologically plausible since the Mediterranean dietary pattern is rich in fiber, antioxidants, including flavonoids, vitamins, carotenoids, and squalene (mainly from virgin olive oil) (20). It may modulate BC risk by decreasing endogenous estrogens (21, 22), increasing sex-hormone-binding globulin levels (23), neutralizing free radicals and preventing DNA damage (24, 25), and reducing oxidative stress (26). Due to the origin and development of Mediterranean diet, it would be expected to find more people accomplishing this lifestyle in Southern Italy than in Northern Italy, partially justifying the different BC incidence rate in these regions. Nevertheless, although there are recognized scores for quantifying adherence to such a diet, it is difficult to establish exactly what such a diet comprises (18).

The present retrospective observational study investigated the relationship between BMI and tumor characteristic of BC patients attending two oncology units, respectively in the Northern and Southern Italy, where a major adhesion to the Mediterranean lifestyle could be presumed.

## Materials and Methods

### Population and Study Procedures

We performed a retrospective analysis on BC patients treated in two different hospitals (Lecce, located in the Southern Italy and Modena, located in the Northern Italy) from January 2019 to December 2020. Lecce was selected as a typical area of Mediterranean basin and Modena as a typical area reflecting the lifestyle of continental Europe. We collected information on 507 BC patients in the Lecce Hospital, whereas 477 cases referred to the Modena Breast Unit, have been used as control group.

Of the 1,071 patients, we excluded seven BC from Modena and 87 from Lecce because of the lack of BMI information. Overall, 976 BC cases were analyzed for the purpose of this study. We calculated BMI as weight in kilograms divided by height in square meters. BMI was categorized according to the World Health Organization (WHO) standard. Four groups were divided according to BMI at the BC diagnosis: underweight, BMI <18.5 kg/m^2^; normal weight, BMI ≥ 18.5–24.9 kg/m^2^; overweight, BMI ≥ 25.0–29.9 kg/m^2^; and obesity, BMI ≥30.0 kg/m^2^.

### Measures of Prognostic Factors

HER2/neu testing was carried out in a single pathology laboratory in Modena and Lecce by immunohistochemistry, and the results were scored as follows: 0, 1 = negative, 2 = indeterminate, and 3 = positive. Patients with human epidermal growth factor receptor-2 (HER2) test results reported as “indeterminate” were evaluated by fluorescence *in situ* hybridization (FISH). Estrogen receptor (ER) and progesterone receptor (PgR) testing were conducted with a single report format of “positive” or “negative” test results, as measured by immunohistochemical analysis (clone 6F11, Ventana, for ER; and clone 1E2, Ventana, for PgR) and staining by Ventana Benchmark autostainer. ER and PgR receptor status were tested by evaluating the percentage of nuclear immunoreactivity with respect to all the nuclei in the neoplastic cells, independently of the staining intensity. Nuclear staining ≥1% of either ER or PgR was considered a positive result. Ki-67 labeling index was determined with the MIB1 monoclonal antibody as nuclear immunoreactivity. The cutoff was equal to 20% to subdivide high and low proliferation index.

*BRCA* test was performed by the Next-Generation Sequencing (NGS) multigene panel technique named SOPHiA Hereditary Cancer Solution (HCS), a CE-IVD-certified application able to identify simultaneously Single Nucleotide Variants (SNVs), indels, and Copy Number Variants (CNVs) in all the 26 tested genes, including *BRCA1* and *BRCA2* (https://www.sophiagenetics.com). Variants were reported using the international standard Human Genome Variation Society (HGVS) nomenclature and classified into five categories: pathogenic (P), likely pathogenic (LP), variant of uncertain significance (VUS), likely benign (LB), and benign (B), according to the American College of Medical Genetics and Genomics (ACMG) criteria (27).

### Statistical Analysis

The tumor characteristics and clinical outcomes of patients after BC diagnosis were analyzed. We collected primary and adjuvant treatment from medical records: surgery, radiation, chemotherapy, and hormonal therapy. We examined a number of potential confounding variables including age at onset, pathological stage, tumor size at surgery, lymph node evaluation at surgery, ER or PgR expression, HER2 expression, pathological subtype, and histological grade. Patients with only biopsy but without surgery were also included.

We compared continuous variables performing the Kolmogorov-Smirnov test and Mann-Whitney *U* test; categorical data were compared using Pearson’s chi- square test or median test, and dicotomic data were compared by odds ratio. Results are reported as median with range or percentages. Percentage has been calculated on valid date. The case-only odds ratio (OR) was used to evaluate the relative strength of the association among BMI and age (<70 *vs.* ≥70 years), diameter (T1 *vs.* ≥T2), breast surgery (quadrantectomy *vs.* mastectomy), and axillary surgery (sentinel lymph node biopsy *vs.* dissection).

Statistical analysis was carried out using the statistical package for social sciences (SPSS 26.0.0; SPSS Inc., Chicago, IL, USA). Statistical significance was set at *p*-value <0.05.

## Results

Nine hundred seventy-seven BC patients were included in the analysis. Overall, 470 were from Modena and 507 from Lecce; the age ranged from 28 to 97 years, with a mean of 61.75 (+/13.06). No differences were observed in the mean age of BC patients from Modena (61.42) and Lecce (62.08). The clinicopathological features are summarized in **Table 1**. Overall, no statistical differences between the two populations were shown in terms of tumor characteristics (ER and PgR expression, HER2 overexpression, and proliferation index) and pathological stage. In detail, pathological tumor size (pT) and pathological lymph nodes (pN) were evaluated. The first information was available for 734 patients, 373 from Modena and 361 from Lecce, while the last information was available for 762 patients, 370 from Modena and 392 from Lecce. Lymph node involvement, pathological tumor size, histological grade, ER and PgR status, HER2 status, and proliferation index were not significantly different among these two groups. By separately analyzing the pathological tumor size as continues variable, we found that Modena BC patients had smaller tumor size (mean dimension of 1.57 cm) compared with Lecce BC patients (mean dimension equal to 1.68 cm) (*p* < 0.001).

**Table 1:** Clinical and biological characteristics of BC patients.

Number of patients (977)	Modena	Lecce	*p-*Value
	470	507	
**Mean age at diagnosis (year)**	61.42 (28–88); SD 12.48	62.08 (28–97); SD 13.65	0.461
**Age group (year)**			
<40	19 (4%)	22 (4.3%)	0.315
40–49	75 (16%)	91 (18%)	
50–69	234 (49.8%)	222 (43.8%)	
>70	142 (30.2%)	172 (33.9%)	
**ER**			
Negative (0)	70 (15.4%)	84 (16.9%)	0.571
Positive (≥1)	385 (84.6%)	414 (83.1%)	
**PgR**			
Negative (0)	91 (21.4%)	128 (25.8%)	0.114
Positive (≥1)	335 (78.6%)	368 (74.2%)	
**HER2**			
Negative	387 (85.1%)	432 (88.5%)	0.116
Positive	68 (14.9%)	56 (11.5%)	
**Proliferation index**			
Low	281 (64.9%)	294 (59.3%)	0.078
High	152 (35.1%)	202 (40.7%)	
**pT**			
pT1	303 (81.2%)	308 (83.3%)	0.139
≥pT2	70 (18.8%)	53 (14.7%)	
**pN**			
pN0	261 (70.5%)	261 (66.6%)	0.240
≥pN1	109 (29.5%)	131 (33.4%)	
**Mean diameter (cm)**	1.57 (0.01–18); SD 1.57	1.68 (0.10–9.9); SD 1.50	<0.001

ER, estrogen receptor; PgR, progesteron receptor; HER2, human epidermal growth factor receptor-2; pT, pathological tumor size; pN, pathological lymph nodes; SD, stardard deviation.

As shown in **Table 2**, data regarding surgical treatments of 920 patients were collected: 450 from Modena and 469 from Lecce. At the Modena hospital, 374 (83.3%) BC patients received a conservative treatment, while 77 (16.7%) received mastectomy. On the other hand, at the Lecce hospital, 469 BC patients were treated by surgery, of whom 322 (68.7%) received a conservative surgery and 147 (31.3%) received a mastectomy. Moreover, at the Modena Hospital, 355 BC patients (84.2%) received sentinel lymph node biopsy (SLNB), 67 (15.8%) axillary dissection, and 28 were not treated in axilla. At the Lecce Hospital, 371 (74.3%) received the SLNB, 58 (25.7%) axillary dissection, three patients were not eligible to be treated by surgery, and 39 did not receive axillary treatment. The surgery adopted between the two groups was statistically different both in the breast and in the lymph nodes (*p* < 0.001).

**Table 2:** Surgical treatment of BC patients.

Surgery	Modena	Lecce	*p-*Value
**Breast surgery**			
Quadrantectomy	374 (83.3%)	322 (68.7%)	<0.001
Mastectomy	77 (16.7%)	147 (31.3%)	
**Lymph node surgery**			
SLNB	355 (84.2%)	371 (74.3%)	<0.001
Dissection	67 (15.8%)	58 (25.7%)	

SLNB, sentinel lymph node biopsy.

A stratified analysis of BMI at diagnosis was conducted and is reported in **Table 3**. Overall, the mean BMI at baseline was 26.88 ± 5.6 kg/m^2^ (overweight). A statistical difference of BMI between the BC patients coming from Modena and Lecce (25.87 and 27.81, respectively; *p* = 0.000001) was found. More overweight and obese patients were found in the Lecce BC group than in the Northern area. Stratifying the population by age, we observed that BC patients coming from Lecce, aged ≥70 years, were more obese compared with the ones from Modena (*p* = 0.000002).

**Table 3:** BMI at baseline of BC patients.

Breast cancer patients’ BMI	Modena	Lecce	*p-*Value
**Mean BMI at the diagnosis (kg/m^2^)**	25.87 (15–48); SD 5.07	27.81 (18–51);SD 6.00	0.000001
**BMI group (kg/m^2^)**			
Underweight <18.5	16 (3.4%)	4 (0.8%)	0.000109
Normal weight ≥ 18.5–24.9	214 (45.5%)	183 (36.1%)	
Overweight ≥ 25.0–29.9	136 (28.9%)	166 (32.7%)	
Obese ≥30	104 (23.4%)	154 (30.4%)	

BMI, body mass index.

With regard to the *BRCA* test, only 210 tests were performed, based on Italian recommendation for the implementation of BRCA testing in breast cancer patients and their relatives (28). The positive rate was 13.1% and 15.1%, respectively, for Modena and Lecce patients. No statistically significant differences were found among the groups of BMI, although a very high rate of *BRCA1* mutation was shown in the obese BC from Lecce. The results are reported in **Table 4**.

**Table 4:** BRCA test and relationship with BMI in Modena and Lecce BC patients.

Breast cancer patients’ BRCA test (210)	Modena		Lecce	
	99		111	
	*N* (%)		*N* (%)	
**BRCA+ 30**	**13 (13.1%)**		**17 (15.1%)**	
**BMI group (kg/m^2^)**	**BRCA1+**	**BRCA2+**	**BRCA1+**	**BRCA2+**
Underweight <18.5	1	2	0	0
Normal weight ≥ 18.5–24.9	3	2	4	4
Overweight ≥ 25–29.9	1	2	2	0
Obese ≥30	1	1	7	0

BMI, body mass index.

The characteristics of the final study population, subdivided by age range and BMI, are reported in **Table 5**. Patients ranging between 40 and 49 years felt in the normal weight group, among 50–69 years were overweight, with a trend toward the obesity for patients from Lecce, and ≥70 years were obese in the Lecce BC group compared with the overweight patients in the Modena group (*p* = 0.0002).

**Table 5:** BMI by age at the diagnosis (kg/m^2^) of BC patients.

Group age	Modena	Lecce	*p-*Value
<40	23.80 (15–38); SD 5.37	24.29 (20–35); SD 3.96	0.698
40–49	24.73 (18–37); SD 5.16	24.20 (18–40); SD 4.32	0.157
50–69	26.21 (15–41); SD 5.06	27.93 (18–51); SD 6.12	0.066
>70	26.34 (17–48); SD 4.81	30.03 (20–48); SD 5.73	0.0002

BMI, body mass index; SD, standard deviation.

**Table 6** describes the characteristics of over 70 BC patients. Of the 314 patients, 307 had information for the clinical characteristics of the tumor as ER, 137 from Modena and 170 from Lecce, while 301 (131 from Modena and 170 from Lecce) had information about the PgR status. The information about the HER2 status was not indicated in one patient aged >85 and one with comorbidity from Lecce and was not collected in seven patients from Modena. Proliferation index was collected for 302 patients, 133 from Modena 169 from Lecce. By categorizing, as dichotomous variables, ER and PgR status, HER2 status, and proliferation index, no significant statistical differences were found among these two groups.

**Table 6:** Tumor characteristic in BC patients aged 70 or more.

Biological parameters	Modena	Lecce	*p-*Value
	149 (%)	172 (%)	
**ER**			
Negative (0)	16 (11.7%)	16 (9.4%)	0.519
Positive ≥1	121 (88.3%)	154 (90.6%)	
**PgR**			
Negative (0)	24 (18.3%)	31 (18.2%)	0.932
Positive ≥1	107 (81.7%)	139 (81.8%)	
**HER2**			
Negative	121 (89.6%)	148 (88.1%)	0.650
Positive	14 (10.4%)	20 (11.9%)	
**Proliferation index**			
Low	96 (72.2%)	119 (70.4%)	0.615
High	37 (27.8%)	50 (29.6%)	
**Mean diameter** (cm)	1.77 (0.10–18.0); SD 2.109	1.84 (0.10–9.9); SD1.657	0.040
**Range of tumor dimension**			
<1 cm	94 (71.8%)	87 (57.6%)	0.014
>2 cm	37 (28.2%)	64(42.4%)	

ER, estrogen receptor; PgR, progesterone receptor; HER2, human epidermal growth factor receptor-2; SD, standard deviation.

Aiming at evaluating if overweight and obesity groups were associated with larger size tumors, we also investigated the dimension of the tumor in centimeters, observing that BC patients from Modena had smaller tumor size with a mean dimension of 1.77 cm, compared with BC patients from Lecce (1.84 cm) (*p* = 0.040). In a logistic regression analysis among age, diameter, breast surgery, axillary surgery, and BMI, only the age ≥70 years and tumor size (>1.5 cm) were significantly associated to the obesity, compared with the normal weight patients (*p* = 0.012 and *p* =0.001, respectively) whereas mastectomy and axillary dissection results were not related to BMI (**Table 7**).

**Table 7:** Case-only ORs and 95% CIs from logistic regression models of the associations among age, T size, breast surgery, axillary surgery, and BMI.

BMI groups	Age			Diameter			Breast surgery			Lymph node surgery		
	<70 *vs.* ≥70			<1.5 *vs.* ≥1.5			Quadrantectomy *vs.* mastectomy			SLNB *vs.* dissection		
	OR	95% CI	*p-*Value	OR	95% CI	*p-*Value	OR	95% CI	*p-*Value	OR	95% CI	*p-*Value
UW	0.146	0.019–1.118	0.064	0.461	0.143–1.490	0.196	2.557	0.815–8.021	2.557	0.343	0.41–2.844	0.321
NW	1.00	Reference	NA	1.00	Reference	NA	1.00	Reference	NA	1.00	Reference	NA
OW	1.349	0.976–1.865	0.070	1.037	0.756–1.422	0.822	0.685	0.976–1.865	0.070	1.066	0.671–1.695	0.786
Ob	1.536	1.100–2.395	0.012	1.726	1.245–2.395	0.001	0.683	0.448–1.041	0.076	1.566	0.981–2.500	0.060

OR, odds ratio; CI, confidence interval; BMI, body mass index; SLNB, sentinel lymph node biopsy; UW, underweight; NW, normal weight, OW, overweight; OB, obese; NA, not applicable.

## Discussion

As already demonstrated by numerous articles, high BMI correlates with BC cancer risk in postmenopausal women (29, 30). Data on women attending the Mammographic Screening Program at Modena, Italy, clearly showed that obese women had a significantly higher incidence of BC (relative risk (RR), 1.32; *p* = 0.040), larger tumors (27% of tumors were larger than T2 size), and more nodal involvement (38.5% of tumors were node positive) than normal-weight women (31). In the meantime, the studies that evaluated influence of overweight and obesity on BC survival have yielded mixed findings (31–33). Obese BC patients diagnosed at the Modena hospital that underwent lifestyle intervention for 1 year, showed a BMI decrease and a statistically significant better outcome than overweight patients who did not obtain a BMI reduction (34).

Thus far, obesity and overweight should be a goal to be defeated by a healthy lifestyle, such as using the Mediterranean diet that includes not only specific eating habits but also PA, exercise, and positive thoughts. Since the Mediterranean diet was developed in Southern Italy and then exported all around the world for its beneficial implications, it would be expected to observe a difference in the BMI distribution between BC patients affected in the Northern and Southern Italy, respectively. Surprisingly, the results of our exploratory analysis show that the rate of obese and overweight BC women was very high along all the Italian country. Indeed, no differences in tumor characteristics and disease stage were found in both BC groups, but an increased BMI rate and a relationship with larger pathological tumor size was found in Southern BC patients aged ≥70 years. Data on larger BC tumor size occurring in obese patients have been demonstrated by several studies compared with normal-weight women (35, 36). A possible reason for these findings is that a lower BMI represents less breast volume, and therefore, earlier discovery of breast masses. This could explain the higher proportion of patients with early-stage breast cancer with a low BMI. The largest tumor size found in Lecce BC patients could also reflect the increased number of mastectomies compared with conservative surgical treatments, associated with more axillary dissections, although no statistically significant relationship was found between obesity and radical surgery. However, data on postmenopausal obese women undergoing conservative surgical treatments plus radiotherapy did not show an increased risk of local regional relapse, not justifying the unnecessary mastectomies in those patients (32, 37).

Of interest, a very high rate of *BRCA1* mutations was found in the obese BC patients from Lecce compared with the same BMI group coming from Modena. The association between obesity and *BRCA* mutation was already hypothesized by Pettapiece-Phillips et al. showing an increase in *BRCA1* mRNA expression with increasing levels of physical activity (PA) that could normalize the protein levels, contributing to stem cell homeostasis and mitigating the effects of an inherited *BRCA* mutation (38). Preliminary evidence from *in vivo* studies and from epidemiologic reports suggest that obesity may affect *BRCA* penetrance through several mechanisms, including insulin resistance and insulin-like growth factor I (IGF-I) regulation. Women with *BRCA1/2* mutations more frequently develop type-2 diabetes after a BC diagnosis compared with carriers without cancer (39). The prediabetic condition, when the levels of insulin and growth factors are typically very high, can raise the risk of BC in these women. A randomized prospective trial with a Mediterranean Diet intervention in *BRCA1/2*-mutated carriers was performed showing an IGF-I and other potential modulators of BRCA penetrance reduction (40).

In our logistic regression analysis, the BMI maintains an independent role as risk factor in older patients (≥70 years), where hyperinsulinemia, which increases adipokines and inflammatory markers that may upregulate cellular proliferation, is more frequent and associated with increased circulating estrogens from adipose tissue (41). Conversely, in premenopausal women, estrogen is mainly derived from the ovaries, rendering less significant the BMI for BC risk (42, 43).

The increased BMI in older BC patients living in Southern Italy (Lecce) was unexpected, since those women were born in the cradle of the Mediterranean lifestyle. It is likely that along the last 70 years, a lifestyle change was taken over, due to the incorporation of new cultural and culinary elements, such as less PA and more refined foods. Furthermore, the economic well-being also provided an increased food amount that contributes to the negative impact of the Mediterranean diet benefits. As recently underlined by the UNESCO, it is important to classify populations as highly or poorly adhering to a Mediterranean diet based on the quantity of food consumed (44). On the other hand, more attention to the physical appearance has been paid by the young generations, particularly in the industrialized regions and then in Northern Italy. In a recent interview performed among North−western Italian population, aimed to investigate the PA profile during the COVID-19 lockdown, 61.8% of interweaved people were moderately active, being tendentially young people more active than adults or elderly (45). On the contrary, data reported by the Italian Institute of Statistics show inactivity levels of nearly 50% among populations of southern Italy, with only about 20% of older adults practicing continuous PA (46). Benefits of an active lifestyle are nowadays well recognized by the WHO that promotes to reaching a goal of 150 min/week of moderate-to-vigorous intensity PA such as walking, running, cycling, or swimming although, in the major industrialized countries, PA rates seem to decline instead of increasing (47). A difference in PA activity could have generate this BMI inversion between the Northern and Southern Italy habits, increasing the rate of BC incidence, particularly at age ≥70 years.

Our study presents several limitations. First, the Mediterranean diet was only presumed for women living in Southern Italy, but no instruments or questionnaire were employed to assess the adherence to a specific diet or to evaluate the grade of PA in these two populations. Second, other risk factors for BC, such as pregnancies, breastfeeding, age at menarche and menopause, or hormonal replacement therapy intake were not evaluated. For all these reasons, our analysis should be only intended as hypothesis generating with the aim to discuss possible lifestyle interventions for the reduction of BC risk.

It is likely that the populations of most countries around the Mediterranean do not consume the Mediterranean diet anymore, and other diets have spread throughout the Italian peninsula. The Nordic diet is based on fruits and vegetables but enriched in omega-3 fatty acids, but a systematic review of literature data showed a weak association between BC incidence and omega-3 fatty acid consumption (48). The ketogenic diet, another modern type of diet that emphasizes on a low intake of carbohydrates, a moderate intake of protein, and a high intake of fat, failed in preventing BC due to the high fat level that promotes the cancer cell progression (49). Only the vegan diet, centered around fruits, vegetables, legumes, and grains, ideally avoiding processed foods and being very similar to the Mediterranean diet, could impact on the BC risk reduction (50).

Interestingly, several nutraceutical compounds, like tocotrienols and unsaturated vitamin E analogs found in several natural sources, such as palm oil, rice bran, and annatto seeds could be useful to counteract obesity and overweight and therefore prevent BC. Two large, randomized studies of alpha-tocopherol supplementation showed a reduction in total cancer rates and in BC onset, particularly associated to a specific genotype of the catechol-O-methyltransferase (COMT) gene (51). Once again, these results underline the growing need to join pharmacogenetic and nutrients aiming to personalize the diet for each one.

Recently, a huge number of data have been provided on the relationship between diet and gut microbiota, postulating an anticancer effect as a result of the composition change of several intestinal bacteria (*Bacillus*, *Enterococcus*, *Lactobacillus*, *Clostridium*, and *Ruminococcus*). The gut microbiota analysis is performed on fecal samples by the Next-Generation Sequencing of variable regions V2–V4–V8 and V3–V6/7–V9 of the codifying gene for RNA 16S. Analyses into specific fecal genera modulated by dietary pattern indicated significantly higher abundance of *Lactobacillus*, *Clostridium*, *Faecalibacterium*, and *Oscillospira* genus and lower abundance of *Ruminococcus* and *Coprococcus* genus bacteria in Mediterranean diet consumers when compared with other kinds of diet (52). A perspective study on gut microbiota analysis could be carried on by BC patients with the aim to evaluate biodiversity according to diet and BMI.

However, greater efforts must be made to restore original habits among Italian women, for instance, by providing Mediterranean cooking lessons and gymnastic courses, which will be the next purpose of our interventional study on BC patients.

## Data Availability Statement

The raw data supporting the conclusions of this article will be made available by the authors, without undue reservation.

## Ethics Statement

Ethical review and approval was not required for the study on human participants in accordance with the local legislation and institutional requirements. Written informed consent for participation was not required for this study in accordance with the national legislation and the institutional requirements.

## Author Contributions

LaC, AT, AI, and MF participated in the conception and design of the study. GRG, FD, and LuC performed the statistical and bioinformatics analyses. LM and MAB provided clinical information. LaC, AT, AI, and MF coordinated, drafted, revised, and finalized the manuscript. All authors contributed to the article and approved the submitted version.

## Funding

This paper was supported by the grant n° 10004 from Angela Serra Association for Cancer Research that provided a financial support for the manuscript publication.

## Conflict of Interest

The authors declare that the research was conducted in the absence of any commercial or financial relationships that could be construed as a potential conflict of interest.

## Publisher’s Note

All claims expressed in this article are solely those of the authors and do not necessarily represent those of their affiliated organizations, or those of the publisher, the editors and the reviewers. Any product that may be evaluated in this article, or claim that may be made by its manufacturer, is not guaranteed or endorsed by the publisher.
